# Targeted Therapies for Pancreatic Cancer: Overview of Current Treatments and New Opportunities for Personalized Oncology

**DOI:** 10.3390/cancers13040799

**Published:** 2021-02-14

**Authors:** Cédric Leroux, Georgia Konstantinidou

**Affiliations:** Institute of Pharmacology, University of Bern, 3010 Bern, Switzerland; cedric.leroux@pki.unibe.ch

**Keywords:** pancreatic ductal adenocarcinoma, therapies, DNA repair, tumor microenvironment, epigenetic alterations, key mutations, autophagy, immunotherapy

## Abstract

**Simple Summary:**

The lack of early diagnosis and the absence of suitable biomarkers coupled with resistance to available therapeutic options render pancreatic cancer one of the deadliest cancer types. Therefore, new therapeutic approaches are needed to be developed, taking into account the genetic and molecular profile of pancreatic tumors. Here, we critically review past and current efforts that have resulted in the development of potent and specific antitumor compounds that, if employed in the appropriate combination therapy, may change this recalcitrant cancer type into a manageable one.

**Abstract:**

Cytotoxic chemotherapy remains the only treatment option for most pancreatic ductal adenocarcinoma patients. Currently, the median overall survival of patients with advanced disease rarely exceeds 1 year. The complex network of pancreatic cancer composed of immune cells, endothelial cells, and cancer-associated fibroblasts confers intratumoral and intertumoral heterogeneity with distinct proliferative and metastatic propensity. This heterogeneity can explain why tumors do not behave uniformly and are able to escape therapy. The advance in technology of whole-genome sequencing has now provided the possibility of identifying every somatic mutation, copy-number change, and structural variant in a given cancer, giving rise to personalized targeted therapies. In this review, we provide an overview of the current and emerging treatment strategies in pancreatic cancer. By highlighting new paradigms in pancreatic ductal adenocarcinoma treatment, we hope to stimulate new thoughts for clinical trials aimed at improving patient outcomes.

## 1. Introduction

Pancreatic ductal adenocarcinoma (thereafter PCa) remains one of the deadliest malignancies with a 5-year overall survival (OS) of only 9% in 2020 [1]. The reason for this lies on the fact that, due to the late diagnosis, about 80% of patients arriving to the clinic have already locally advanced and unresectable PCa as a result of local invasion of adjacent structures. Based on the tumor stage at the time of diagnosis, PCa can be treated with surgery, chemotherapy, radiation therapy, and targeted therapy with different recommendations [2,3,4]. At a resectable PCa setting, surgery can have a curative (when all the tumor can be removed) purpose. According to recent clinical practice guidelines of the American Society of Clinical Oncology, modified FOLFIRINOX (folinic acid, 5-fluorouracil, irinotecan and oxaliplatin, thereafter mFOLFIRINOX) must be the preferred adjuvant therapy for patients with pancreatic adenocarcinoma who have undergone an R0 or R1 resection and have not received prior neoadjuvant chemotherapy [5]. The term “modified” refers to the reduction of irinotecan from 180 to 150 mg/m^2^ and the exclusion of the 5-FU bolus due to the emergence of adverse effects of FOLFIRINOX. Moreover, in the adjuvant/post-operative setting, conventionally fractionated radiation is recommended for patients with high-risk features such as positive lymph nodes and margins. For patients with locally advanced and metastatic disease (unresectable PCa), systemic chemotherapy, generally mFOLFIRINOX or gemcitabine/nab-paclitaxel combination, followed by radiation therapy is recommended, and depending on the presence of alterations, including specific genetic mutations, mismatch repair deficiency, or high microsatellite instability, may receive additional targeted therapies. However, in such advanced stage and due to the aggressive cell biology of PCa with continuing therapy resistance, the available treatment options are not sufficient for curative outcomes. While chemotherapy clearly improves the OS of PCa patients at the preoperative and postoperative setting, radiotherapy is subjected to controversy due to conflicting clinical trial results and its association with a narrow therapeutic index. The impact of radiotherapy in the management of PCa has been extensively reviewed elsewhere [6].

Aside from the general aging of our society, obesity and type-2 diabetes play a role in the etiology of PCa. In this case, a chronic low-grade inflammation may be a potential mechanism linking obesity to increased PCa incidence and progression [7,8,9]. Moreover, lifestyle habits, including alcohol abuse and tobacco use appear to contribute to PCa development [10]. Lastly, there are some genetic syndromes characterized by specific mutations such as *BRCA1*, *BRCA2* (Breast and ovarian cancer syndrome)*, ATM* (Ataxia telangiectasia), *STK11* (Peutz-Jeghers syndrome)*, PRSS1* (hereditary pancreatitis), *MLH1,* and MSH2/6 (Lynch syndrome) that are associated with PCa for a subgroup of patients, representing additional risk factors [11]. A deeper understanding of the pathology of PCa may explain therapeutic resistance, survival differences, and responses to specific therapies. The genetic landscape of PCa is characterized by somatic mutations in one or more of the four major genes: *KRAS, CDKN2A, TP53,* and *SMAD4* [12]. Besides these mutations, the development of PCa depends on the tumor microenvironment (TME). Targeting single deregulated pathways is often ineffective owing to redundant signaling and complex crosstalk. Moreover, the high degree of inter-tumoral genetic heterogeneity of PCa suggests that it is unlikely that a single targeted therapy will work [13,14]. Therefore, a combination of druggable key signaling hubs needs to be identified and targeted. In this review, we critically summarize the latest targeted combination therapies for advanced/metastatic PCa and discuss new viewpoints for therapeutic approaches currently under preclinical evaluation.

## 2. Methods

To search for clinical trials, we used the https://www.clinicaltrials.gov (accessed on 20 December 2020) database. We selected for randomized clinical trial studies for PCa patients with reported results (339 studies). From these, we excluded overlapping studies, studies not aimed for combination therapies, and studies for resectable PCa. Moreover, the terminated trials without results are mentioned in the context only if the reason for termination was lack of efficacy and/or toxicity. In addition, when appropriate, we refer to some ongoing clinical trials specially for new targeted therapies, i.e., chimeric antigen receptor T cells (CAR-T) due to the lack of finalized trials.

## 3. Targeting DNA Repairing Deficiency and Microsatellite Instability

Genomic instability is a key feature of almost all human cancers [15]. Such modifications benefit the clonal growth of cancer cells, including improvements in gene copy numbers, rearrangements, and mutations. Nevertheless, these same defects often produce cancer cell vulnerabilities that could be used for anticancer therapies. A substantial population of PCa patients harbors germline or somatic mutations in genes that are involved in the DNA damage repair (DDR) pathway, such as BRCA1/2 and ATM [16], suggesting that these patients may benefit from personalized targeted therapies. Moreover, PCa cells may be selectively sensitive to DDR inhibitors because KRAS mutations, which are present in 95% of PCa cases, are associated with increased replication stress due to depletion of nucleotide pools [17] and the slowing of replication fork activity [18].

### 3.1. PARP Inhibitors

Poly (ADP-ribose) polymerase (PARP) enzymes detect and bind to single-strand DNA breaks (SSB), leading to the recruitment of hundreds of proteins to repair the SSBs. However, if SSBs are not repaired, they cause stall of replications forks and eventually progress to double-strand breaks, which are highly cytotoxic to cells. Thus, cancer cells with mutations that prevent homologous recombination repair, such as BRCA1/2 loss-of-function mutations, are often synthetically lethal with PARP inhibitors due to significantly lower DDR [19]. A phase II clinical trial with olaparib, a small molecule PARP inhibitor, in a small cohort of *BRCA1/2* mutated advanced PCa patients with gemcitabine resistance, evidenced a tumor response rate of 50.0% (4/8 patients), while 25% showed stable disease ≥8 weeks with a median overall survival (OS) of 9.8 months [20]. Similarly, a phase III clinical trial with olaparib that included 154 metastatic PCa patients with a germline *BRCA1* or *BRCA2* mutation resulted in a median progression-free survival (PFS) of 7.4 months in the olaparib group vs. 3.8 months of the placebo group [21]. Thus, the documented efficacy of PARP inhibitors in PCa patients with germline *BRCA1* or *BRCA2* mutation underscores the importance of germline testing for all patients with PCa.

In addition to PARP inhibitors alone, several trials are currently underway to evaluate PARP inhibitor combinations with other classes of therapies causing DNA damage in PCa patients. However, in >80% of patients, PARP inhibitors, when combined with chemotherapy, including olaparib plus gemcitabine and olaparib plus irinotecan or cisplatin, showed substantial toxicity [22,23]. Clinical trials designed to assess the effectiveness of certain PARP inhibitor combinations of chemotherapy and/or dose-reduced chemotherapy will help decide whether or not such combinations will play a role in providing therapeutic efficacy in PCa patients.

### 3.2. ATM Inhibition

In contrast to PARP inhibitors, the use of other DDR inhibitors is currently restricted to early clinical studies. Ataxia Telangiectasia Mutated (ATM) is a serine/threonine kinase involved in DDR signaling and it is one of the most commonly mutated DDR genes, with a number of somatic or germline mutations identified in PCa [24,25]. A mouse model of ATM deficient PCa evidenced an increased number of pancreatic intraepithelial precursor lesions, fibrosis, and a greater degree of epithelial to mesenchymal transition compared to the control mice, suggesting a role in PCa progression [26]. ATM inhibitors such as AZD0156 in combination with olaparib, irinotecan, or fluorouracil in patients with advanced solid tumors are currently in phase I clinical trials (NCT02588105). Cancer cells may compensate the loss of ATM by upregulating ATR, indicating that ATR inhibitors may display efficacy in ATM-deficient tumors, including PCa. However, ATM-deficient PCa cell lines undergo cell death only when incubated with olaparib plus AZD6738, an ATR inhibitor, but neither agent alone [27]. Thus, patients with ATM deficient PCa may benefit from combination therapies targeting PARP and ATR.

Intriguingly, recent preclinical data showed that the addition of DNA-PK inhibitors with PARP and ATR inhibitors provide synergistic antitumor effects in both ATM-deficient and ATM-proficient cells [28]. If this approach turns out to be feasible in the clinic, it will considerably extend the target population that can benefit from such combination therapies.

### 3.3. ATR Inhibition

ATR is one of the primary targets of DDR inhibitors since both SSBs and double-strand DNA breaks (DSBs) are the main regulatory features of ATR [29]. There are currently five ongoing clinical trials assessing ATR targeting compounds: AZD6738 (NCT02630199, NCT03669601), M6620 (NCT03718091), M4344 (NCT02278250), and BAY1895344 (NCT03188965). In PDAC cell lines, AZD6738 inhibited gemcitabine-induced Chk1 activation, prevented cell cycle arrest, and strongly induced replication stress. Interestingly, the combination of AZD6738 and gemcitabine induced tumor regression in a subgroup of tumors in the KRAS^G12D^; p53^R172H^; Pdx-cre (KPC) mouse model in vivo [30]. Similar to human PCa, KPC mouse tumors are known to be refractory to therapy, suggesting that the combination of ATR with chemotherapy may be effective in a subset of human PCa patients. M6620 is the first ATR inhibitor tested for monotherapy and combined with various chemotherapies, including topotecan, carboplatin, gemcitabine, and cisplatin [31,32,33]. M6620 monotherapy was well tolerated without observation of dose limiting toxicities and the combination with carboplatin showed clinical activity in patients with advanced solid tumors [33]. Nevertheless, chemotherapy combinations were associated with higher rates of bone marrow toxicity which required M6620 dose reduction. Thus, when used in combination with systemic DNA damaging chemotherapy, it is necessary to optimize the dose/frequency of ATR inhibitors to allow normal tissue recovery. More ongoing phase I clinical trials aim at determining the safety and maximum tolerated dose of ATR inhibitors in combination with chemotherapy in patients with advanced solid tumors. Of these, NCT02630199 and NCT03669601 are testing AZD6738 in combination with paclitaxel and gemcitabine, respectively, while the NCT02278250 aims at testing M4344 in combination with carboplatin.

### 3.4. DNA-PK Inhibition

The DNA-dependent protein kinase (DNA-PK) is involved in the non-homologous end joining (NHEJ) pathway [34]. This class of drugs is particularly important in combination with ionizing radiation (IR) because NHEJ is the prevailing repair mechanism for IR-induced DNA double-strand breaks [35]. Indeed, DNA-PK genetic deficiencies sensitize cells to IR and other DSB-inducing agents [36]. M3814 (peposertib), a highly potent and selective inhibitor of DNA-PK, sensitized pancreatic cancer cells to IR in vitro and provided complete tumor regression upon treatment with IR in vivo [37]. In the clinical setting, M3814 has been tested in a phase I clinical trial (NCT02316197) in which it was well-tolerated when given orally as a single agent in doses up to 400 mg BID [38]. Based on the promising preclinical and clinical trials, M3814 has progressed into a phase I/II clinical testing in combination with hypofractionated radiation therapy for the treatment of unresectable locally advanced PCa (NCT04172532). This study, which is currently recruiting participants, is expected to be concluded in 2024.

### 3.5. CHK1/2 Inhibition

Cell cycle progression is monitored by mechanisms that control the transition from quiescence (G0) to proliferation, ensuring genetic transcript fidelity. Checkpoint kinase 1 (CHK1) and CHK2 are serine/threonine protein kinases that are part of the recognition of DNA damage. CHK1 is an important signal transducer and the trigger of G2 checkpoint activation, while CHK2 is involved in the repair of DNA, cell cycle, and apoptosis in DDR. *CHEK2* gene mutations have been identified in a wide variety of cancers, including PCa [39]. A CHK1/2 inhibitor, AZD7762, alone or in combination with gemcitabine significantly sensitized PCa cells (MiaPaCa-2) to radiation. Interestingly, the radiosensitization was associated with abrogation of the G2 checkpoint, inhibition of Rad51 focus formation, inhibition of homologous recombination repair, and persistent gamma-H2AX expression [40]. However, AZD7762 in combination with gemcitabine provided only a partial objective response in gemcitabine-naïve patients (NCT00413686). Moreover, AZD7762 was found to be cardiotoxic, which occurred at doses as low as 30 mg and had to be stopped [41,42].

A phase I/II trial with rabusertib (LY2603618), a highly selective CHK1 inhibitor, assessed whether combination with gemcitabine could prolong OS compared to gemcitabine alone in 99 patients with unresectable PCa (NCT00839332). The results from this study evidenced that the combination of rabusertib with gemcitabine did not confer a greater survival advantage compared to gemcitabine alone [43]. Recently, another preclinical study showed that prexasertib (LY2606368), a drug currently in phase I clinical trials, increases the sensitivity of PCa cells to gemcitabine and S-1 (an orally available fluoropyrimidine derivative) [44]. Prexasertib is currently being evaluated in combination with olaparib (NCT03057145) or multiple other targeted drugs (NCT02124148) in advanced solid tumors.

Overall, the lack of obvious clinical efficacy and the reported increased cardiotoxicity warrant further studies to clearly assess whether CHK1 inhibitors can be used for PCa therapy.

### 3.6. Wee1

The Wee1 protein kinase phosphorylates CDK1^Tyr15^, resulting in G2/M checkpoint activation [45]. Thus, Wee1 inhibition prevents initiation of G2 checkpoints, causing transformed cells with damaged DNA to go through mitosis and cell death. There are multiple completed or ongoing clinical trials with Wee1 inhibitors in many tumor types that have been extensively reviewed by Ghelli Luserna di Rorà et al. [46]. One of these clinical trials examined AZD1775, a Wee1 kinase inhibitor, as monotherapy or in combination with chemotherapy (gemcitabine, cisplatin, or carboplatin) in patients with refractory solid tumors (NCT00648648). Of 176 patients that were given combination therapy, 94 (53%) had stable disease and 17 (10%) achieved a partial response. Interestingly, the response rate in *TP53*-mutated patients (*n* = 19) was 21% compared with 12% in *TP53* wild-type patients (*n* = 33) [47]. In PDAC, AZD1775 was tested in a dose escalation study alone or combined with gemcitabine (+radiation) in a cohort of 34 patients with locally advanced unresectable PCa (NCT02037230). In this trial, the combination of AZD1775 with gemcitabine and radiation resulted in an OS of 22 months compared to the 11.9 to 13.6 months of gemcitabine/radiation alone [48]. Currently, the benefits of adding the AZD1775 into a gemcitabine + nanoparticle albumin-bound (nab)-paclitaxel are being evaluated in a phase I/II clinical trial in patients with previously untreated unresectable or metastatic PCa (NCT02194829).

Preclinical evidence has indicated that Wee1 inhibitors show synergistic effects when combined with histone deacetylase (HDAC) inhibitors [49], proteasome inhibitors [50], tyrosine kinase inhibitors [51], anti-apoptotic protein inhibitors (enhance dependency on BCL-2 and/or MCL-1 inhibition) [52], and mammalian (or mechanic) target of rapamycin (mTOR) inhibitors [53]. This latter study is particularly noteworthy for PCa as mTOR inhibition was found to synergize with Wee1 inhibition in KRAS mutant tumors. Future research is necessary to assess whether the combination of Wee1 with mTOR inhibitors may be an effective therapeutic strategy for the treatment of PCa.

## 4. Targeting Epigenetic Alterations

Despite particular mutations in DNA or loss of genes, the rate of gene expression is regulated by complex mechanisms controlling access to DNA and transcription functionality. Notably, epigenetic changes are considered essential for the initiation and progression of PCa as well as resistance to therapy [54].

### 4.1. miRNA

MicroRNAs are non-coding RNAs that interact with mRNA leading to its degradation or reduced translation [54]. miRNAs regulate and are regulated by a number of key pathways that involve cell differentiation, proliferation, and apoptosis [55]. In PCa, many miRNAs are consistently upregulated (miR-21, miR-155, and miR-221), while others are downregulated (miR-34, miR-200 family, miR-15a, miR-506, miR-96, miR-145, miR-155) compared to healthy pancreatic tissue. Moreover, the discovery that miRNAs are detectable in blood (miR-21, miR-155, miR-196a, miR-221) [56,57], pancreatic juice (miR-21, miR-155) [58,59] or stool samples [60] suggests that miRNAs can be used as biomarkers in PCa. Indeed, miRNAs are not only differentially expressed in PCa compared to healthy tissue, but their expression is also strongly associated with PCa staging. In a study with 47 PCa patients evaluating the correlation between plasma miR-221 concentrations and clinicopathological factors, the patients with high plasma miR-221 concentration showed positive correlation with the presence of distant metastasis and advanced (non-resectable) cancer status [61].

Accumulating evidence suggests that miRNAs by modulating key targets and pathways such as KRAS, PI3K/AKT, TP53, NF-κB, and Hedgehog signaling are associated with resistance of PDAC to chemotherapy. In gemcitabine-treated PDAC cells, the expression of miR-21 dampens the anti-tumor activity of gemcitabine. Moreover, evidence suggests that miR-21 inhibits the tumor suppressor gene, phosphatase and tensin homologue (PTEN), thereby activating the PI3K/AKT pathway. Intriguingly, in human PCa tissue, miR-21 overexpression correlates with worse outcome of patients treated with gemcitabine [62]. Similarly, Li et al. showed that miR-200b, miR-200c, and let-7 family (let-7b, let-7c, let-7d, let-7e) are down-regulated in gemcitabine-resistant PCa cells. Accordingly, restoration of miR-200 and let-7 resulted in increased PCa sensitivity to gemcitabine [63]. Moreover, miR-365 overexpression induced gemcitabine resistance by directly targeting the adaptor protein Src homology 2 domain of 1 (SHC1), as well as BAX protein promoters of apoptosis [64]. Overall, the re-expression or inhibition of miRNAs seems to be an effective strategy for the treatment of PCa, yet more advanced pre-clinical and clinical studies are required to better understand the potential of miRNAs modulation for PCa therapy.

### 4.2. DNMTs

The DNA methyltransferase 1 (DNMT1) is required for DNA methylation during replication [65]. Zagorac et al. found that pancreatic cancer stem cells (CSCs) evidenced hypermethylation via DNMT1 upregulation. Pharmacologic or genetic targeting of DNMT1 in CSCs reduced their self-renewal and in vivo tumorigenic potential [66]. Interestingly, aberrant hypermethylation begins at early stages of PanINs and its incidence progressively increases during neoplastic development; therefore, DNMT1 inhibitors are under intense clinical investigation [67,68]. Decitabine (a DNMT1 inhibitor) is being tested in combination with gemcitabine for the treatment of refractory PCa and the study is expected to complete in early 2021 (NCT02959164). Moreover, there is an ongoing clinical trial testing decitabine in combination with anti-PD-1 antibodies and chemotherapy in relapsed or refractory malignancies (NCT02961101). Lastly, a new nucleoside analog, 5-aza-4-thio-2-deoxycytidine (Aza-TdC), has been shown to decrease DNMT1 levels and suppress tumorigenesis in lung tumor xenografts [69]. Aza-TdC is currently in phase I clinical trials in patients with advanced solid tumors (NCT03366116).

### 4.3. HATs and HDACs

Targeting the pattern of histone acetylation with either histone deacetylase (HDAC) or histone acetyl transferases (HAT) inhibitors result in increased or decreased histone acetylation, respectively. HDAC inhibitors induce hyperacetylation of histones and thus reactivate tumor suppressor gene expression, leading to suppression of cell proliferation, cell differentiation, and apoptosis [70]. Although the HAT inhibitors are still in pre-clinical phases, the HDAC inhibitors have progressed into clinical assessment and there have been many clinical phase I/II trials over the years. A phase II randomized clinical study with CI-994, an oral HDAC inhibitor, and gemcitabine in patients with advanced PCa (NCT00004861) did not show greater efficacy compared to gemcitabine alone [71]. Vorinostat, a HDAC inhibitor, was assessed in phase I trials together with capecitabine and radiation therapy in 21 patients with non-metastatic PCa (NCT00983268). The most common adverse events that were observed with vorinostat were lymphopenia (76%) and nausea (14%), suggesting that the drug is overall well tolerated. However, despite an encouraging median OS, the small number of patients enrolled in this trial prevented the proper evaluation of this therapy [72]. Moreover, the treatment with panobinostat for patients that progressed on gemcitabine-based therapy (NCT01056601) or advanced solid tumors (NCT00550199) had to be terminated because of a complete lack of treatment response and early treatment-related toxicity [73]. Thus, HDAC inhibitors seem to have serious adverse effects, requiring careful clinical evaluation. Moreover, patient stratification based on transcriptome or epigenetic signatures is essential for the correct assessment of these targeted therapies.

### 4.4. Bromodomain Proteins

The limited success of HDAC inhibitors in clinical trials against solid tumors increased the interest in targeting epigenetic “readers” of histone acetylation, in particular members of bromodomain (BRD) and extra terminal domain (BET) family of proteins. These proteins recognize acetylated lysine residues and regulate molecular interactions with relevance to transcriptional control. In PCa, BRD4 was found to promote cell proliferation, enhance gemcitabine resistance [74], and block the proliferation of PCa cells in three-dimensional collagen [75]. Moreover, BET inhibition decreased desmoplastic pancreatic stromal cell proliferation and suppressed the growth of patient-derived pancreatic tumor xenografts (PDX) by inhibiting Hedgehog and TGF-β pathways [76]. I-BET762, a benzodiazepine compound, inhibited PDAC cell proliferation and enhanced the therapeutic effect of gemcitabine in Panc1-derived xenograft tumors [77]. However, in clinical trials, many BET inhibitors showed adverse effects and lack of efficacy in solid tumors [78]. Molibresib (GSK525762) was tested in 19 patients with nuclear protein in testis (NUT) midline carcinomas (NCT01587703). NUT midline carcinoma is a rare and invariably lethal cancer caused by the fusion protein BRD4-NUT, which spurs faulty gene expression [79]. Molibresib was well tolerated, and out of the 19 patients, 4 achieved partial response, 8 had stable disease as best response, and 4 were progression-free for more than 6 months, providing a promising future for this inhibitor [80]. MK-8628, an oral BET inhibitor targeting BRD2 and BRD4 is in phase Ib clinical trial in patients with selected advanced solid tumors, but 38/46 (83%) patients manifested treatment-related adverse events (NCT02259114) [81]. Moreover, another clinical study of 13 patients with MK-8628 was terminated due to limited efficacy (NCT02698176).

In summary, epigenetic approaches to PCa therapy have considerable potential but face challenging clinical translation due to poor response and treatment-related adverse events. In order to effectively implement this clinical approach, it is important to first determine the tumor subtypes and specific cancer vulnerabilities to tailor specific drug combinations.

## 5. Targeting Key Signaling Pathways

### 5.1. KRAS and Main Targets

**Figure 1:**
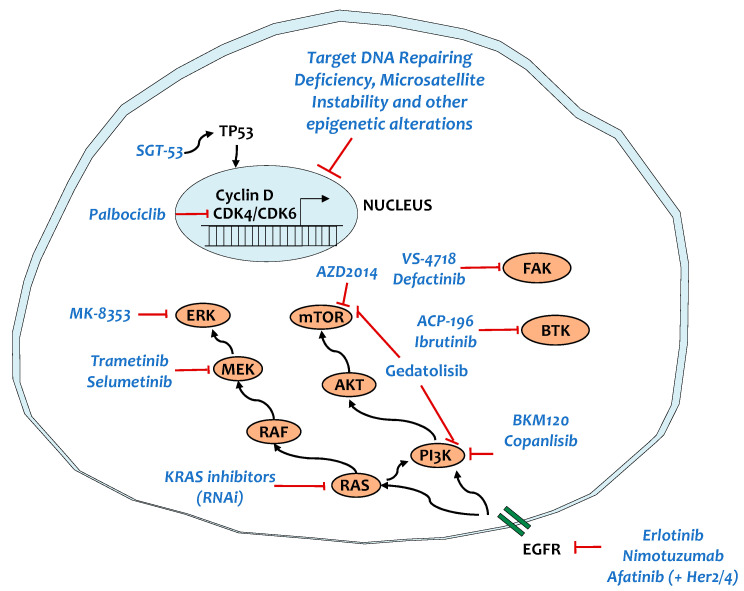
Targeting key signaling pathways in patients with pancreatic ductal adenocarcinoma (PCa). EGFR, epidermal growth factor receptor; RAS, rat sarcoma viral oncogene; AKT, protein kinase B; CDK4/CDK6, cyclin-dependent kinase 4/6; RAF, rapidly accelerated fibrosarcoma; MEK, mitogen-activated protein kinase; ERK, extracellular signal-regulated kinases; PI3K, phosphoinositide 3-kinase; mTOR, mechanistic target of rapamycin; FAK, focal adhesion kinase; BTK, Bruton’s tyrosine kinase.

KRAS is mutated in >90% of PDACs [82]. This high prevalence has contributed to considerable interest in therapies that selectively target mutated KRAS. Inhibitors targeting KRAS^G12C^ are currently in clinical development (i.e ARS-1620 and AMG 510), showing promising results in colorectal and lung cancers [83,84]. However, KRAS^G12C^ mutations are rare in PCa (~1% of all KRAS mutations), excluding them as a therapeutic option. Because inhibitors that specifically target the most prevalent KRAS mutations in PCa do not exist, there is a lot of interest in developing means to deliver small interfering RNAs (RNAi) in vivo via exosomes or small extracellular vesicles (Figure 1). This strategy has recently entered a phase-I trial for metastatic PCa patients and is expected to be completed in early 2022 (NCT03608631).

Another approach that is currently being tested to target mutated KRAS is with peptide-based vaccines. This approach is described in the context of the “vaccine therapy” section below.

In view of the difficulty to directly target KRAS, during the last four decades efforts were focused on developing treatments aimed at targeting its main downstream effector pathways, including the RAS-RAF-MEK-ERK and the PI3K-AKT-mTOR signaling pathways (Figure 1). However, in patients with metastatic PDAC, no clinical benefit was observed with MEK inhibitors plus gemcitabine compared with gemcitabine alone (NCT01016483, NCT01231581) [85,86]. Despite these deceptive findings, recent research has helped to clarify a potential resistance mechanism of PCa to MAPK inhibition. Indeed, results by two different research groups showed that pharmacological inhibition of the ERK or MEK signaling pathway in KRAS-mutant PCa elicited a protective increase of autophagy [87,88]. Moreover, related to MAPK inhibition, a recent study revealed that combination MEK1/2 and CDK4/6 inhibitors triggers senescence-associated secretory phenotype (SASP)–mediated increase in CD31^+^ cells and endothelial activation, promoting CD8^+^ T cell tumor infiltration in PCa [89]. Consequently, the addition of gemcitabine or anti-programmed death-1 (anti-PD-1) therapy to the above-described combination resulted in tumor regression, suggesting that combining senescence-inducing therapies with both chemotherapy and immunotherapy may be an effective strategy to treat PCa. However, care should be taken to first assess the impact of this therapeutic regiment in metastatic PCa because of the increase in CD31^+^. Even though it suppresses the primary tumor, it may contribute to the metastatic potential.

There are multiple alterations affecting the PI3K pathway in PCa. PCa has been shown to harbor activating mutations in *PIK3CA* (~4%) and/or loss of the tumor suppressor PTEN (25–70%) [82,90]. Moreover, increased AKT activity has been identified in about 60% of PCa samples and amplification of *AKT2* occurs in 10–20% of the cases [91]. This mutational profile, together with the highly promising preclinical antitumor efficacy of the PI3K pathway inhibitors in combination with clinically relevant interventions [92,93,94], provided the rationale for the initiation of clinical trials. A first clinical trial with Copanlisib (BAY 80-6946), a potent pan-class I PI3K inhibitor, showed promising anti-tumor pharmacodynamic activity and clinical benefit in patients with advanced solid tumors, including PCa (NCT00962611) [95]. In parallel, BKM120, a pan-class I PI3K inhibitor, was tested in combination with mFOLFOX6 (5-FU/leucovorin plus oxaliplatin) in metastatic PCa patients (NCT01571024). Unfortunately, this combination resulted in increased toxicity compared with that expected from either the PI3K inhibitor or the chemotherapy alone [96]. Similarly, increased toxicity was found when BKM120 was given in combination with the MEK inhibitor, trametinib (GSK1120212), in patients with advanced BRAF/KRAS mutant solid tumors (NCT01155453) [97]. This toxicity was somehow expected as also other PI3K or AKT or dual PI3K/mTOR and MEK inhibitor combination studies have shown similar toxicity, with fatigue, gastrointestinal, and cutaneous toxicity being most predominant [97,98,99]. Currently, there is an ongoing clinical trial with a dual PI3K/mTOR inhibitor, gedatolisib (PF-05212384), in combination with a CDK4/6 inhibitor, palbociclib (PD-0332991), for patients with advanced solid tumors including PCa. These trials will assess the safety and efficacy of these compounds in order to determine whether these combinations can be applied in PCa patients.

Lastly, there are multiple trials focusing on mTOR inhibitors (Figure 1). AZD2014, a potent dual mTORC1 and mTORC2 inhibitor showed promising preclinical results in the KPC (*LSL-Kras^G12D^*; *Trp53^R172H^; Pdx1-Cre*) mouse model. However, the first phase I clinical study with AZD2014 evidenced that, out of the 56 patients, only 2 showed confirmed partial response. Interestingly, one patient with confirmed partial response was a patient with acinar PCa bearing *KRAS*, *PDGFRA*, *APC*, *ERB4*, *KIT,* and *FBXW7* mutations [100]. AZD2014 was also tested in a cohort of 27 patients with TSC1/2 mutated refractory solid cancer as monotherapy (NCT03166176). However, the study was withdrawn due to lack of efficacy. AZD2014 is currently undergoing evaluation in combination therapies in patients with solid tumors, including one with the Bcl-2 inhibitor, navitoclax (NCT03366103) or with selumetinib (AZD6244), a MEK1/2 inhibitor (NCT02583542).

### 5.2. TP53

Preclinical research has focused on restoring/reactivating wild-type p53 or to destabilize mutant p53 [11,101]. Statins recently showed a promising potential in depleting mutant p53 via inhibition of the mevalonate pathway [102]. Nevertheless, either ineffective or sluggish clinical translation of targeting p53 was achieved [103]. The safety of SGT-53, a liposomal formulation encapsulating a plasmid carrying a human wild-type p53, has been recently evaluated in a phase I trial for the treatment of solid tumors (NCT00470613) [104]. This trial not only showed that systemically delivered SGT-53 is well tolerated and exhibits anticancer activity, but also provided evidence of targeted delivery of SGT-53 to metastatic lesions. A phase II trial is currently carried out in combination with gemcitabine/nab-paclitaxel and SGT-53 for metastatic PCa and is expected to be finalized at the end of 2021 (NCT02340117).

### 5.3. SMAD4

SMAD4 mutations are widespread in PCa patients (detected in 16–44% of patients) [105]. Loss of SMAD4 accelerates PCa progression and correlates with metastasis and poor prognosis [106]. Recent evidence suggested that metformin inhibits PCa progression and improves the survival of patients with SMAD4-deficient PCa, while this is not the case in patients with SMAD4-normal PCa [107]. These findings suggest that metformin could be used to selectively treat patients with SMAD4 deficiency and should be further investigated in the clinical setting.

### 5.4. Tyrosine Kinase Signaling

Tyrosine kinases (TKs) play an important role in malignancy [108]. They are able to initiate a range of intracellular signals that govern proliferation, cell survival, transformation, differentiation, migration, and metastases [109,110]. Compared to other mutant KRAS tumors, such as lung cancer, the activation in TKs is not a common future of PCa, yet targeting TKs was proved to prevent the insurgence of resistance mechanisms [111]. The epidermal growth factor receptor (EGFR) inhibitor, erlotinib, is one of the only targeted drugs with significant survival benefit in the clinic that received approval for the treatment of patients with metastatic PCa (Figure 1). Erlotinib treatment prolongs the survival of PCa patients partly by blocking gemcitabine-induced MAPK signaling activation [112]. Nimotuzumab, which is a humanized EGFR-optimized antibody, increased the 1-year survival rate of PCa patients by 2-fold in a phase II trial randomized (gemcitabine plus nimotuzumab vs. gemcitabine plus placebo) [113]. Oddly, the patients carrying wild type KRAS showed better survival compared to those carrying mutant KRAS.

The *ERBB2* (*HER2*) gene is frequently amplified (24%) in PCa, and it is associated with worse prognosis partly by mediating resistance to gemcitabine and irinotecan/SN-38 treatment [114,115]. Afatinib, an irreversible EGFR, HER2, and HER4 small molecule inhibitor, is currently being evaluated in clinical studies in combination with capecitabine (NCT02451553) or selumetinib (AZD6244), a MEK1/2 inhibitor (NCT02450656) for the treatment of advanced stage PCa or with gemcitabine/nab-paclitaxel for metastatic PCa (NCT02975141).

Focal adhesion kinase (FAK) is a non-receptor TK with a role in invasion, growth, and metastasis, which is found elevated in high-grade mutant KRAS non-small cell lung cancer and PCa [116,117]. In PDAC, FAK interacts with both tumor and stromal cells and its activity levels are associated with immunosuppressive TME and poor survival. The administration of a FAK inhibitor (VS-6063) in combination with gemcitabine and nab-paclitaxel has been shown to delay tumor growth in PDX models compared to chemotherapy alone [118]. Moreover, a FAK inhibitor (VS-4718) in the KPC mouse model (p48-CRE; LSL-KRas^G12D/wt^; p53^flox/wt^) decreased fibrosis, TAMs, myeloid-derived suppressor cells (MDSCs), regulatory T (Treg) cell infiltration, and improved mice survival [117]. However, the signal transducer and activator of transcription 3 (STAT3) signaling-triggered compensatory survival pathways and rendered tumors resistant to FAK inhibition [119]. Given the role of FAK in modulating the TME, a clinical trial is in progress to examine the interaction of FAK inhibitors with immune checkpoint inhibitors (defactinib/pembrolizumab: NCT02758587). Moreover, another ongoing trial is currently evaluating whether reprograming the tumor microenvironment by targeting FAK following chemotherapy can potentiate the anti-PD-1 antibody-mediated anti-tumor response (NCT03727880).

The non-receptor TK, Bruton’s tyrosine kinase (BTK), plays a role in proliferation of leukemic cells in many B-cell malignancies [120]. The BTK inhibitor, ibrutinib, has already shown clinical activity in hematological malignant patients. In mouse models of PCa, administration of ibrutinib contributed to the reprogramming of M2 macrophages to an antitumor M1 phenotype that boosted CD8+ T cell cytotoxicity and suppressed PCa growth [121]. A combination therapy has been carried out with acalabrutinib (ACP-196), a BTK inhibitor, in combination with pembrolizumab (an FDA approved checkpoint inhibitor targeting PD-1) in patients with advanced or metastatic PCa (NCT02362048) [122]. The results from this trial evidenced that although the combination of acalabrutinib and pembrolizumab was well tolerated and peripheral reduction of MDSCs was observed, the overall response rate was limited for both regimens, with responses in 0% of patients undergoing monotherapy and 7.9% of those receiving combination therapy.

## 6. Targeting the Tumor Microenvironment and Related Metabolic Reprogramming

The TME of PCa consists of stromal cells, a dense ECM, and immune cells. PCa stroma is a crucial feature, regulating tumor growth, vascularization, drug responsiveness, immune landscape, and metastasis. Indeed, stromal fibroblasts (termed cancer associated fibroblasts, CAFs) play an important role in promoting PCa progression and in dampening chemotherapeutic response [123]. The ECM that occupies the bulk of tumor mass is a dense network of structural and adaptor proteins, proteoglycans, and enzymes [124]. Among these components, hyaluronic acid (HA), a glycosaminoglycan, limits the accessibility of chemotherapeutic agents to cancer cells by increasing interstitial pressure leading to vascular collapse and reduced tumor perfusion [125]. Extensive preclinical research evidenced that recombinant human hyaluronidase 20 (PEGPH20) reduces total intratumoral pressure and vascular collapse [125,126], which encouraged further clinical testing. However, among the trials that have been performed in combination with chemotherapy in metastatic PCa patients provided discouraging outcomes (Table 1). One trial in unselected for tumor HA status patients reported treatment-related grade 3 to 4 toxicity in the PEGPH20 + mFOLFIRINOX arm vs mFOLFIRINOX alone and failed to show any difference in OS [127]. Similarly, a phase III trial of gemcitabine plus nab-paclitaxel with or without PEGPH20 failed to meet the expected primary end point of OS or PFS and had to be terminated (NCT02715804) [128]. Currently there is an ongoing trial of PEGPH20 in combination with pembrolizumab for patients with previously treated, HA-high metastatic PCa (NCT03634332).

Given the rich stromal component of PCa TME, one would expect that eliminating stromal components may be an effective strategy for PCa therapy. However, preclinical studies proved that this is not the case because radical stromal depletion via Hedgehog deletion resulted in more aggressive, poorly differentiated, and highly vascularized tumors [137]. Similarly, complete depletion of αSMA+ fibroblasts during PCa development accelerated disease progression and decreased mouse survival [138]. Importantly, two clinical trials with vismodegib, a Hedgehog pathway inhibitor, in combination with gemcitabine or gemcitabine/nab-paclitaxel in metastatic PCa patients did not improve the overall response rate, PFS, or OS compared to gemcitabine or gemcitabine/nab-paclitaxel alone (Table 1) [129,130]. On the other hand, suppression of the lipid-triggered pro-fibrotic plasminogen activator inhibitor-1 (PAI-1) pathway limited desmoplasia and immunosuppression, and delayed tumor progression without enhancing tumor vascularization in preclinical KPC and orthotopic mouse models of PCa [139]. These results suggest that limiting some stromal components rather than completely eliminating them may help to gradually allow therapeutic agents to reach tumors without risking enhancing metastases formation. Currently, there are many ongoing clinical trials targeting different aspects of stromal cell biology, which have been extensively summarized by Hosein and colleagues [140].

The previously described desmoplastic environment of PCa limits the availability of nutrients and oxygen in cancer cells. Indeed, PCa is highly hypoxic, with median pO2 < 5.3 mmHg compared to the surrounding normal pancreatic tissue where the median pO2 reaches 24.3–92.7 mmHg [141]. Moreover, due to the already highly hypoxic environment of PCa, anti-angiogenic therapy is not an option as worsens tumor hypoxia, promoting chemoresistance, cancer stem cell enrichment, invasion, and metastasis [142,143]. Indeed, chronic treatment with an anti-vascular endothelial growth factor (anti-VEGF) antibody in a mouse PCa model (*LSL-Kras^G12D^; Cdkn2a^lox/lox^; p48^Cre^*) increased collagen deposition, epithelial plasticity, and metastasis [144]. Accordingly, a series of phase III clinical trials with VEGF inhibitors, such as bevacizumab (a humanized anti-VEGF-A monoclonal antibody), axitinib, or sorafenib with various drug combinations (gemcitabine or gemcitabine/erlotinib) in patients with advanced or metastatic PCa failed to improve OS (Table 1) [131,132,133,134].

**Figure 2:**
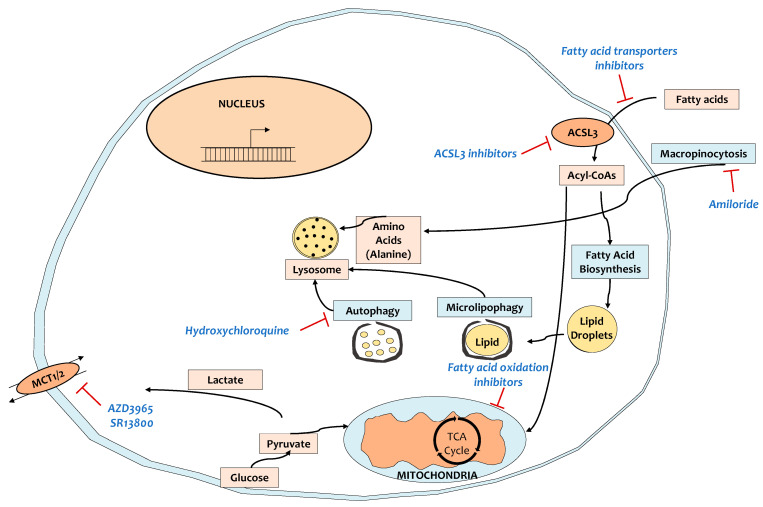
Targeting metabolic reprogramming in patients with PCa. KRAS-mutant tumors rely on metabolic reprogramming, exposing critical vulnerabilities to target for therapy. MCT1/2, monocarboxylate transporters 1/2; ACSL3, acyl-CoA synthetase long-chain 3.

In absence of oxygen and nutrients, cancer cells must rely on several metabolic rearrangements for survival such as a shift from oxidative phosphorylation to glycolysis, inhibition of fatty-acid desaturation, increased scavenging of lipids and proteins and high basal autophagy (Figure 2) [145,146,147,148,149]. An acute effect of hypoxia is the shift from oxidative phosphorylation to glycolysis due to hypoxia inducible factor 1a (HIF1a) stabilization [150]. As the glycolytic shift of hypoxic tumors could be considered as a targetable vulnerability, attempts have been made to develop inhibitors that would target the glycolytic pathway; 2-deoxy-D-glucose (2DG) is a synthetic glucose analog and phase I clinical trials in solid tumors evidenced that 2DG can be safely combined with docetaxel because the adverse effects were tolerable (NCT00096707) [151]. However, since then, there have been no new clinical trials assessing 2DG in combination with other compounds. Another promising approach would be to target lactate transporters (monocarboxylate transporters: MCTs). MCT1/2-selective inhibitors (AZD3965) are currently being tested in advanced tumors (NCT01791595). However, one patient developed malignant hyperlactaemic acidosis upon treatment with AZD3965 due to a preexisting metabolic disorder (hyper-Warburgism) [152]. Thus, patients with elevated plasma lactate must be excluded from the treatment with MCT inhibitors (Figure 2).

KRAS-transformed cells scavenge extracellular proteins through macropinocytosis as a major source of amino acids, which are the source of the central carbon metabolism and tricarboxylic acid cycle [147,148]. Inhibition of macropinocytosis by amiloride inhibited in vitro PCa cell proliferation and tumor growth in vivo [153,154], suggesting that that may be an interesting strategy in clinical trials for PCa therapy (Figure 2). Similarly, KRAS-mutant tumors rely on scavenging of extracellular lipids [149,155,156], which is a potential vulnerability. Indeed, we showed that inhibition of acyl-CoA synthetase long chain 3 (ACSL3), an enzyme that activates extracellularly-derived fatty acids by adding a CoA moiety, delayed tumor growth, suppressed fibrosis, and enhanced the activation and abundance of CD8+ T cells in KPC mice [139].

Autophagy is a catabolic process of degradation in which autophagosomes engulf macromolecules and specific organelles and then fuse with lysosomes to provide the cell with recycled building blocks and substrates for metabolism and energy (Figure 2) [157]. PCa cancer cells have been shown to actively use high autophagy to survive and proliferate [158]. The antimalarial drug chloroquine inhibits autophagy and suppresses the proliferation of PCa cells in vitro, leading to xenografted mouse tumor regression [158]. In the pre-operative setting, the combination of hydroxychloroquine (HCQ) with gemcitabine/nab-paclitaxel evidenced improved tumor and serum biomarker response (CA 19-9) and enhanced tumor immune cell infiltration compared to gemcitabine/nab-paclitaxel alone [135]. Despite these promising trials, preliminary results of a similar ongoing study in metastatic PCa patients (NCT01506973) showed that the addition of HCQ to gemcitabine/nab-paclitaxel did not improve the primary endpoint of overall survival at 12 months [136], suggesting that more rationalized approaches must be followed (Table 1). For instance, upon certain interventions, autophagy has been shown to be highly activated acting as a pro-survival mechanism [87,88], indicating that, in some contexts, autophagy inhibitors may be particularly successful.

## 7. Targeting Immune Regulatory Networks

**Figure 3:**
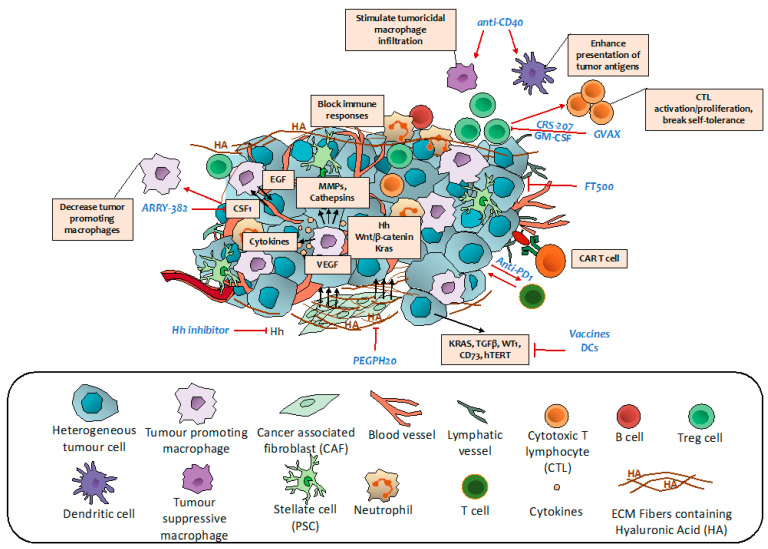
Targeting immune regulatory networks in PCa patients. Some of the current actionable interventions for pancreatic cancer.

The induction of an immune response against tumors in many advanced-stage cancers has been proven to be extremely effective. However, like with other antitumor strategies, PCa is highly refractory. The reason for this is that the TME of PCa is occupied by an impressive number of highly immunosuppressive cell populations, namely tumor associated macrophages (TAMs), myeloid-derived suppressor cells (MDSCs), neutrophils, and regulatory T cells (Tregs) that allow immune evasion and limit the effectiveness of chemotherapy (Figure 3) [159]. Moreover, TAMs not only affect the activity of cytidine deaminase, a key enzyme in gemcitabine metabolism, hence driving resistance to gemcitabine-based chemotherapy [160], but also mediate angiogenesis by releasing cytokines and growth factors such as VEGF [161]. Furthermore, because PCa is known to contain relatively few genomic mutations in protein coding regions, there is a limited number of neoantigens that could be used for immunotherapy. Several immunotherapeutic strategies (vaccination, adoptive cell transfers, and targeting immune checkpoints) are being evaluated in PCa. The choice of the chemotherapeutic drug to combine with immunotherapy has to be carefully considered since many chemotherapeutic drugs suppress immune activation. Chemotherapy that is able to induce immunogenic cell death such as oxaliplatin, cyclophosphamide, and gemcitabine are preferred [162]. For instance, immediately after the initial course of gemcitabine treatment, naïve, activated immune functions are triggered [163]. However, there is also a decline in memory T cells, suggesting that rationalized treatment protocols must be used in clinical trials when combining chemotherapy with immunotherapy.

### 7.1. Vaccine Therapy

Vaccines prime the patient’s own T cells against cancer-specific antigens, promoting effective anticancer immunity. Cancer vaccines which can be whole cell, peptide-, dendritic cell (DC)- or DNA-based, have shown high potential in triggering persistent increase in T cell response, can be easily evaluated for personalized target development and, most importantly, are well tolerated (Table 2).

GVAX pancreas is an irradiated allogeneic whole pancreatic tumor cell vaccine in which cancer cells are engineered to express granulocyte-macrophage colony stimulating factor (GM-CSF). GVAX pancreas combined with low dose cyclophosphamide to inhibit Treg cells, and a cancer vaccine, CRS-207 (live, attenuated *Listeria monocytogenes* expressing mesothelin), which stimulates innate and adaptive immunity in previously treated metastatic PCa, improved OS of patients compared to historical OS achieved with chemotherapy (NCT01417000) [179]. However, when a phase IIb trial was performed in a bigger cohort of previously treated metastatic PCa patients with cyclophosphamide + GVAX + CRS-207 the OS did not improve compared to chemotherapy (Table 2) [164].

Regarding peptide-based vaccination, administration of a cocktail containing mutated RAS peptides combined with GM-CSF in advanced PCa patients showed vaccine-induced immune response—32% of the patients had stable disease after peptide vaccination and all of the patients with stable disease showed an immunological response, while 45% of patients showing an immune response to the vaccine had progression of disease [165]. This triggered a second study in which previously resected PCa patients, treated as above (but with a pool of 7 peptides against mutant RAS) were followed up for 10 years [166]. This study showed the persistence of T cells recognizing vaccine peptides many years after the last vaccination (Table 2). However, in a pilot study, out of the 9 evaluable patients that were vaccinated with a 21-mer peptide containing the corresponding KRAS mutation of the patient’s tumor, only 1 patient showed immune response [180]. In this case, the extremely low number of patients, coupled with the inconsistent number of vaccinations performed, may have limited this study. Taken together, these results suggest that cancer peptides targeting mutant KRAS should be included in the standard of care of all PCa patients, but before this, carefully designed combination therapies need to be performed (Figure 3).

Another tumor-specific antigen that has been tested in PCa is the human telomerase reverse transcriptase (hTERT) with the GV1001 vaccine consisting of 16 amino acids of hTERT. A phase III trial of 1062 patients treated either with gemcitabine/capecitabine alone or concurrently or sequentially with GV1001 provided no survival benefit in patients with advanced PCa. However, T cell proliferation was positive in 31% of patients given sequential immunotherapy and 14.7% of patients given concurrent chemoimmunotherapy, suggesting that sequential immunotherapy should be preferred [167].

Survivin, a member of the inhibitor of apoptosis (IAP) family, is another potentially attractive target. A phase II study in advanced, previously treated PCa patients with SVN-2B, a peptide derived from survivin 2B protein in combination with interferon β (IFNβ), showed longer OS in patients receiving the combination vs SVN-2B alone or placebo [168]. These data also show that IFNβ is a promising adjuvant for peptide vaccination therapy. Moreover, it was subsequently shown that treatment with SVN-2B triggered a dense infiltration of CD8^+^ T cells in some patient lesions and a high rate of programmed cell death ligand 1 (PD-L1) expression in cancer cells, indicating emergence of resistance against CTL attack [181]. This clearly provides a rationale for the combination of SVN-2B with anti-PD1 or anti-PD-L1 therapies.

DCs are powerful antigen presenting cells that can be manipulated in vitro to develop a cancer vaccine in order to increase antigen presentation, break tolerance against tumor-associated antigens where it is lacking, or enhance T cell priming (Figure 3). Unfortunately, the clinical studies performed with DC-based vaccines are only limited to early phase with a small number of patients. Of these, one study used gemcitabine followed by DCs pulsed with the MHC-I, -II, or -I/II–restricted epitopes of Wilms tumor (WT1), which is highly expressed in PCa [169]. Importantly, 50% of the patients showed increased OS, PFS, and specific T cell response.

### 7.2. Monoclonal Antibodies

CD40 activation can reverse immune suppression and drive antitumor T cell responses (Figure 3) [182]. Preclinical evidence in KPC mice (Kras^LSLG12D/+^, Trp53^LSL-R172H/+^, Pdx1-Cre) suggested that CD40 agonist antibody combined with immune checkpoint inhibitors (ICI), trigger tumor regressions, and immunological memory. Of note, this is not the case when ICIs are given alone [183]. The combination of a CD40 agonist antibody with gemcitabine in a small cohort of patients with chemotherapy-naïve, surgically incurable PCa evidenced improved OS and PFS (Table 2) [170]. Moreover, there is an ongoing trial testing the APX005M (CD40 antibody) with gemcitabine and nab-paclitaxel with or without nivolumab (anti-PD-1) in patients with previously untreated metastatic PCa (NCT03214250) or CDX-1140 (CD40 antibody), either alone or in combination with CDX-301 (FLT3L), pembrolizumab or chemotherapy (NCT03329950). Given the previously reported antitumor efficacy of CD40 agonists when combined with ICIs and their capacity to stimulate tumoricidal macrophage infiltration in tumors [170], if safe, these combinations are promising in bringing new hope for PCa treatment.

### 7.3. Immune Checkpoints Inhibitors

The objective of ICIs is to intensify existing anti-cancer responses by improving stimulatory or blocking activity of the immune system regulators to allow better clearing of cancer cells (Figure 3). Monotherapy with ipilimumab (anti CTLA-4) on locally advanced and metastatic PCa did not show any benefit (NCT00112580). Moreover, combination of ipilimumab with gemcitabine did not prove to be more effective than gemcitabine alone in advanced PCa in one phase Ib trial [171]. However, when gemcitabine was combined with 10 or 15 mg/kg tremelimumab, the OS was longer compared to historical data of gemcitabine monotherapy, suggesting that a high dose of ICIs should be obtained for increased efficacy (Table 2) [172]. Interestingly, when ipilimumab was combined with GVAX in patients with previously treated PDAC, 3 out of 15 patients showed stable disease, and 7 out of 15 patients manifested a decline in CA19-9 [173]. Pembrolizumab (anti PD-1) is approved for patients with advanced PCa that harbor high microsatellite instability, DNA mismatch repair deficiency, or high tumor mutational burden. A phase Ib/II trial of a combination of pembrolizumab with gemcitabine/nab-paclitaxel in chemotherapy naïve metastatic patients showed improved efficacy vs historical gemcitabine/nab-paclitaxel [174].

Based on recent preclinical and diagnostic approaches, CD73, a nucleotide metabolizing enzyme that sustains immune homeostasis, is highly expressed in tumor PCa cells and it is associated with poor survival independently of the number of tumor-infiltrating lymphocytes or TNM stage [184]. Clinical trials combining a CD73 Inhibitor, LY3475070 (NCT04148937) or CPI-006 (NCT03454451) alone or in combination with pembrolizumab in patients with advanced cancers, including PCa are currently ongoing. Thus, CD73 may be a novel immunotherapeutic target and a promising immune prognostic biomarker for PCa in the future.

### 7.4. Adoptive Cell Transfer Therapy

Adoptive cell transfer (ACT) therapy uses ex vivo expanded tumor-infiltrating lymphocytes and is the most prominent form of immunotherapy with impressive results in B-cells malignancies and 20–25% of patients with metastatic melanoma [185,186]. Two trials assessing ex vivo-expanded, cytokine-induced killer (CIK) cells in gemcitabine-refractory advanced PCa, reported no OS or PFS improvement in patients compared with previous trials, yet the treatment improved patients’ quality of life (pain, gastrointestinal distress, jaundice, body image alterations, altered bowel habits, health satisfaction, and sexuality) [176]. Related to mutant KRAS targeting, a phase II clinical trial designed to test whether the ACT of ex vivo expanded tumor-infiltrating lymphocytes targeting personalized cancer neoepitopes can mediate regression of metastatic solid cancers, identified CD8^+^ T cells reactive to KRAS^G12D^ (NCT01174121). Expansion and infusion of KRAS^G12D^ reactive CD8^+^ T cells back to a colorectal cancer patient resulted in regression of metastatic lung lesions [187]. If successful, this may be a potential strategy to provide in combination studies for PCa treatment.

Currently, there are many ongoing chimeric antigen receptors (CAR)-T cell therapies for PCa. In a phase I study, patients with chemotherapy-refractory metastatic PDAC, received T cells engineered to transiently express an mRNA encoding a CAR specific for mesothelin (Table 2) [177]. The results from this trial revealed stable disease in 2 out of the 6 patients, underscoring the potential of mesothelin-specific CAR T cell therapy for PCa. Moreover, a phase I/II adoptive T cell trial in 7 locally advanced and metastatic PCa patients using infusions of anti-CD3/anti-EGFR bispecific antibody armed activated T cells, induced anti-cancer cytotoxicity, and increased innate immune responses [178].

Lastly, there are many ongoing clinical studies against multiple PCa relevant targets such as prostate stem cell antigen (PSCA) NCT02744287, carcinoembryonic antigen positive (CEA+) for liver metastases (NCT03818165), and CD133 (NCT02541370). Moreover, new potentially promising combination therapies are ongoing targeting the tumor cell-associated antigen Nectin 4 and FAP present in CAFs in patients with Nectin4-positive/FAP-positive advanced solid tumors (NCT03932565).

### 7.5. Other Immune Regulators

Preclinical studies revealed that colony-stimulating factor-1 (CSF1) and receptor (CSF1R) blockade not only decreases the number of the immunosuppressive TAMs, but also reprograms the remaining ones to support antigen presentation and bolster T cell activation within the tumor microenvironment (Figure 3) [188]. Based on this strong rationale, a trial assessing a CSF1R inhibitor, ARRY-382, in combination with pembrolizumab in patients with advanced solid tumors has been finalized and the results are pending (NCT02880371). Given the role of CSF1/CSF1R blockade in TAM reprogramming, this therapeutic option may be successful in combination therapies.

CXCR-4 is an alpha-chemokine receptor specific for stromal-derived-factor-1 which has been found to cause immune suppression and promote PCa [189]. The combination of CXCR4 antagonist, BL-8040, with pembrolizumab in patients with metastatic PCa, is currently being assessed (NCT02907099 and NCT02826486). Moreover, more trials are underway with plerixafor (CXCR4 antagonist), in combination with cemiplimab (anti-PD-1) in metastatic PCa patients (NCT04177810).

Natural killer (NK) cells have the ability to target and destroy tumor cells without prior sensitization, via activation of NK cell-activating receptors against ligands present on target tumor cells, providing a promising tool in the field of cancer immunotherapy [190]. Currently, there is an ongoing trial with FT500, an induced pluripotent stem cell-derived NK cell product in combination with ICIs (NCT03841110). Although it is early to say, one expected drawback of therapy with NK cells in PCa is their relatively short lifespan, which causes a reduction in in vivo persistence and therapeutic efficacy. Future combination studies will provide information on the possibility to add NK cells in patients’ treatment options.

## 8. The Road to Personalized Oncology

The ideal world of personalized oncology is that of a patient going to the clinic, having an image-guided percutaneous core needle biopsy, and subsequently screened to identify its mutational and transcriptional profiles, which would allow for the optimal design of a personalized treatment regimen. Although pancreatic tumors are considered generally “cold” or non-T cell inflamed, some tumor subtypes called immunogenic display significant immune infiltrate, antigen presentation, CD4^+^, and CD8^+^ T cells and show upregulation of CTLA-4 and PD-1 suppressive pathways [191]. These results suggest that these patients may benefit from immunotherapy with checkpoint inhibitors.

With the emergence of new technologies such as whole genome and single-cell RNA sequencing, there are many ongoing trials assessing the genome and transcriptional profiling of patients with advanced pancreatic tumors to identify predictive biomarkers and new actionable targets for therapy. For example, *GATA6* expression in tumors was found to be a robust biomarker in basal-like tumor subtypes having significantly lower levels compared to classical subtype tumors. Interestingly, the basal-like tumor subtypes were found in the metastatic PCa patients, suggesting that this subtype may be present in more advanced tumors. Interestingly, 20 potentially actionable somatic mutations were found in 30% of the patients assessed involving *ARID1A* (*n* = 8%), *BRAF* (*n* = 2%), *CDK4/6* (*n* = 7%), *PIK3CA* (*n* = 7%), *PTEN* (*n* = 5%), and *RNF43* (*n* = 3%), giving rise to possibilities for personalized treatment for some of these patients [192].

The major limit to the success of personalized oncology for PCa patients is the lack of time from biopsy to treatment due to the fast disease progression that gives only a little time for personalized screening strategies. Moreover, the tumor site differences and the high intratumoral heterogeneity of PCa suggest that an exact treatment protocol may not be possible. There are many actionable targets currently under development (summarized in Table 3), but more rationalized and controlled clinical trials are needed. For instance, many studies described in this review were performed with a very low number of patients and/or lack of adequate controls within the same population. Moreover, there are many safe and potentially promising targeted therapies, yet some studies did not screen the patients for expression of specific targets before treatment, making difficult any interpretation. Since not all patients are positive for a specific tumor associated antigen, every clinical trial should first screen the target population for patients that are destined to respond (i.e mesothelin-high vs low) in order to obtain meaningful results. We hope that in the near future, biopsy-based genomic, transcriptomic, and immune landscape analysis for all PCa patients will become the standard of care.

## 9. Conclusions

Pancreatic ductal adenocarcinoma is a critical and increasing global health concern, hence it is highly essential to improve the effectiveness of the currently available therapeutic options. The complexity and the signaling pathway redundancy of RAS have reduced the successful targeting of RAS-mediated oncogene dependence at the clinical level [193]. Thus, combination therapies are the only option to treat RAS-mutant tumors. Recent experimental evidence made significant progress towards a better understanding of the unique characteristics of PCa progression and in relation to its surrounding microenvironment. From past successes and failures, we learned that an effective therapeutic strategy depends on the timing of its application. This model suggests that tumors should “ask” for a specific intervention and the timing of the addition of a compound may be determinant for the efficacy of a combination therapy.

## Figures and Tables

**Table 1:** Summary of clinical trials modulating the tumor microenvironment and metabolic reprogramming in PCa patients.

Drug	Targeted Pathway	Phase/Patient Number	Intervention	Subject Population	Results	Refs
PEGPH20	HA	Phase 1b/II *n* = 138	mFOLFIRINOX	Metastatic PCa	Grade 3 to 4 toxicity and worsened OS	NCT01959139 [127]
PEGPH20	HA	Phase III *n* = 494	Gem + nab-PTX	Metastatic PCa	Grade 3 to 4 toxicity and worsened OS	NCT02715804 [128]
Vismodegib	Hedgehog pathway	Phase Ib/II *n* = 106	Gem	Metastatic PCa	Does not improve overall response rate, PFS, or OS	NCT01064622 [129]
Vismodegib	Hedgehog pathway	Phase II *n* = 71	Gem + nab-PTX	Metastatic PCa	Does not improve overall response rate, PFS, or OS	NCT01088815 [130]
Bevacizumab	VEGF pathway	Phase III *n* = 535	Gem	Advanced PCa	Does not improve OS	NCT00088894 [131]
Bevacizumab	VEGF pathway	Phase III *n* = 607	Gem + erlotinib	Metastatic PCa	Does not improve OS, improved PFS	NCT01214720 [132]
Axitinib	VEGF pathway	Phase III *n* = 632	Gem	Advanced PCa	Does not improve OS, improved PFS	NCT00471146 [133]
Sorefenib	VEGF, PDGF and RAF pathway	Phase III *n* = 102	Gem	Advanced PCa	Does not improve overall response rate, PFS, or OS	NCT00541021 [134]
HCQ	Autophagy	Phase II/*n* = 98	Gem + nab-PTX	Preoperative PCa	Greater tumor response, improved serum biomarker response, and immune activity	NCT01978184 [135]
HCQ	Autophagy	Phase II/*n* = 112	Gem + nab-PTX	Metastatic PCa	Greater pathological tumor response, but not OS	NCT01506973 [136]

Gem: Gemcitabine; nab-PTX: Nab-paclitaxel; HCQ: Hydroxychloroquine; OS: Overall survival; PFS: Progression-free survival.

**Table 2:** Summary of clinical trials targeting immune regulatory networks.

Drug	Targeted Pathway	Phase/Patient #	Treatment Combo	PCa	Result	Refs
Cancer vaccines						
GVAX	T cell response	Phase IIb *n* = 169	CP + CRS-207	Previously treated metastatic	Well tolerated, does not improve OS vs chemotherapy	NCT02004262 [164]
Peptide vaccine	mRAS	Phase I/II *n* = 38	GM-CSF as adjuvant	Advanced	Durature memory against mRAS, activation of RAS-specific T cells, improved OS vs non responders	CTN-97004 [165]
Peptide vaccine	mRAS	10 year follow-up *n* = 11	GM-CSF as adjuvant	Advanced/lymph node metastases	Durature memory against mRAS (years) positive immune response in all patients	CTN-98010 [166]
Peptide vaccine (GI-4000)	hTERT	Phase III *n* = 1062	Gem+ Cap	Advanced/Metastatic	Well tolerated, does not improve OS	[167]
Peptide vaccine (SVN-2B)	HLA-A24 of survivin 2B	Phase II *n* = 83	IFNβ	Advanced	SVN-2B + IFNβ improved OS and immunological reaction vs placebo	UMIN000012146 [168]
DC-based vaccine	WT1	Phase I *n* = 10	Gem	Advanced	Increased OS, PFS and specific T cell responses.	UMIN00004063 [169]
Monoclonal antibodies						
CD40 monoclonal antibody (CP-870,893)	T cell responses	Phase I *n*= 21	Gem	CT-naïve, unresectable/metastatic	Mild cytokine release syndrome, 4/21 PR, 11/21 SD, and 4/21 PD, improved OS and PFS vs Gem	NCT00711191 [170]
Immune checkpoint inhibitors						
Ipilimumab	CTLA-4	Phase Ib *n* = 16	Gem	Advanced	Grade 3 to 4 hematologic adverse events, 2/16 PR, 5/16 SD, does not improve OS	NCT01473940 [171]
Tremelimumab (CP-675,206)	CTLA-4	Phase I *n* = 34	Gem	Advanced	Grade 3 to 4 hematologic adverse events, improved OS vs historical Gem results	NCT00556023 [172]
Ipilimumab	CTLA-4	Phase I *n* = 30	GVAX	Previously treated, advanced	3/17 SD, 7/15 reduced CA19-9, improved OS vs ipilimumab alone	NCT00836407 [173]
Pembrolizumab	PD-1	Phase Ib/II *n* = 17 (11 evaluable)	Gem + nab-PTX	Metastatic	Grade 3 to 4 hematologic adverse events, 3/11 PR, 8 SD	NCT02331251 [174]
Nivolumab/nab-PTX	PD-1	Phase I *n*= 44	Gem	Advanced	2% CR, 16% PR, 46% SD ≥ 6 weeks, 20% PD	NCT02309177 [175]
Adoptive cell therapy/CAR-T						
Cytokine-induced killer cells	MHC- antitumor activity	Phase II/*n* = 20		Second-line Gem refractory advanced	Does not improve OS or PFS, but improves quality of life	NCT00965718 [176]
CAR-T	Mesothelin	Phase I/*n* = 6		CT-refractory metastatic	SD (2/6)	NCT01897415 [177]
CAR-T	Mesothelin	Phase I/II *n* = 7	anti-CD3 + anti-EGFR	Advanced/Metastatic	Anti-cancer cytotoxicity and increased innate immune responses	NCT02620865 [178]

CP: Cyclophosphamide; Gem: Gemcitabine; CT: Chemotherapy; nab-PTX: Nab-paclitaxel; HCQ: Hydroxychloroquine; OS: Overall survival; PFS: Progression-free survival; CR: Complete response; PR: Partial response; SD: Stable disease; PD: Progressive disease; Combo: Combination; #: means the patient number.

**Table 3:** Summary of mechanisms of oncological treatment.

Targeted Mechanism	Targeted Pathway
DNA Repairing Deficiency and Microsatellite Instability	PARP
	ATM
	ATR
	DNA-PK
	CHK1/2
	Wee1
Epigenetic Alterations	miRNAs
	DNA methyltransferase 1
	HATs and HDACs
	Bromodomain proteins
Key Signaling Pathways	KRAS, PI3K, mTOR
	TP53
	SMAD4
	Tyrosine Kinase Signaling (EGFR, HER2, FAK, BTK)
Tumor Microenvironment and Related Metabolic Reprogramming	HA
	Hedgehog
	VEGF
	Glycolysis, monocarboxylate transporters
	Autophagy
Immune Regulatory Networks	GM-CSF
	Mutated KRAS peptides (T cell response)
	hTERT peptides (T cell response)
	Survivin peptides (T cell response)
	DCs (WT1)
	CD40
	CTLA-4
	PD-1/PD-L1
	CD73
	CD3/EGFR
	PSCA (ongoing)
	CEA+ (ongoing)
	CD133 (ongoing)
	Nectin 4/FAP (ongoing)
	CSF1/CSF1R (ongoing)
	CXCR-4 (ongoing)
	Pluripotent stem cell-derived NK cells (ongoing)
